# A prospective single arm cohort study: An analysis of the effectiveness of surgical treatment of locally advanced breast cancer

**DOI:** 10.1016/j.heliyon.2024.e36279

**Published:** 2024-08-13

**Authors:** Ying Xu, Xin Huang, Chang Chen, Yan Li, Yidong Zhou, Songjie Shen, Yan Lin, Qiang Sun

**Affiliations:** Department of Breast Surgery, Peking Union Medical College Hospital, Chinese Academy of Medical Sciences and Peking Union Medical College No.1 Shuaifuyuan, Dongcheng District, Beijing, 100730, China

**Keywords:** Breast cancer, LABC, Surgery, Survival

## Abstract

**Background:**

Breast cancer stands as a globally significant contributor to both incidence rates and mortality among women. Approximately 10–15 % of women will face a diagnosis of an advanced yet potentially treatable stage of the disease. When individuals diagnosed with locally advanced breast cancer (LABC) exhibit resistance to preoperative chemotherapy and experience tumor progression, they unfortunately forfeit the opportunity for surgical intervention, thereby diminishing the prospects for a radical cure.

**Method:**

We conducted a prospective, single-arm cohort study aimed at evaluating the feasibility of locally modified radical resection for LABC with skin invasion. The primary endpoints encompassed overall survival (OS) and disease-free survival (DFS), whereas the secondary endpoint focused on the quality of life (QoL) among breast cancer patients.

**Results:**

Between March 2018 and December 2022, a total of 38 eligible patients were enrolled in this study. The Kaplan-Meier estimates for 1-year, 3-year, and 5-year DFS among all patients were 69.8 %, 53.3 %, and 37.5 %, respectively. Correspondingly, the OS rates were 100.0 %, 85.6 %, and 68.0 %. Both univariate and multivariate analyses revealed that patients with a history of neoadjuvant chemotherapy who exhibited stable or progressive disease had inferior DFS outcomes. Notably, patients demonstrated clinically meaningful and statistically significant enhancements in functional status and overall QoL. However, no notable improvement was observed in specific symptom domains.

**Conclusion:**

Patients with locally advanced breast cancer, specifically those presenting with T4 tumors, who undergo surgical intervention followed by postoperative adjuvant therapy, can attain favorable prognostic outcomes and experience an enhanced quality of life.

## 1 Background

Breast cancer represents a significant contributor to the incidence and mortality rates among women globally [1]. In 2020, it was estimated that approximately 2.3 million new cases were diagnosed, resulting in 0.69 million fatalities. Projections indicate that the global cancer burden is anticipated to escalate to 28.4 million cases by 2040, marking a 47 % increase from 2020 levels [2]. Notably, 10–15 % of women will receive a diagnosis of locally advanced breast cancer (LABC), a more advanced stage of the disease however remains potentially curable [3].

According to AJCC TNM staging criteria 8th edition [4], locally advanced breast cancer (LABC) encompasses cases where the primary breast tumor measures greater than 5 cm in size, or exhibits involvement of the chest wall and/or skin, along with extensive clinical lymph node involvement, categorized as N2 or N3 disease status. These patients are generally designated as having non-resectable breast cancer, a state that implies a grim prognosis despite the absence of distant metastases. In the absence of localized therapeutic measures, these individuals are at risk of developing complex local sequelae, including ulceration, intractable pain, and opportunistic infections, all of which can severely impair the quality of life for patients with breast cancer. The utilization of precise medical terminology underscores the importance of timely diagnosis and specialized treatment protocols to manage this challenging clinical scenario [5]. Currently, comprehensive preoperative therapies, encompassing neoadjuvant chemotherapy, aim to reduce tumor burden and potentially render initially inoperable breast cancers amenable to surgical resection. Breast cancer patients with T4 tumors are identified as eligible candidates for preoperative systemic therapy, in accordance with NCCN guidelines. Nonetheless, if patients with locally advanced breast cancer (LABC) exhibit insensitivity to preoperative chemotherapy, resulting in tumor progression, they may forfeit the opportunity for surgical intervention and, consequently, the potential for radical tumor eradication. This underscores the critical need for individualized treatment strategies and close monitoring of treatment response in this patient population.

The localized treatment option for breast cancer, exemplified by modified radical mastectomy, is characterized by minimal surgical trauma and expedited patient recovery. Subsequently, comprehensive therapeutic modalities, which encompass adjuvant radiotherapy and chemotherapy, can be initiated as early as two weeks post-surgery, ensuring a seamless transition into the next phase of treatment [6]. In clinical practice, we have observed that patients diagnosed with locally advanced breast cancer (LABC) who undergo surgical resection followed by postoperative adjuvant therapy experience significant improvements in quality of life and favorable prognostic outcomes. Irrespective of prior neoadjuvant therapy administration, surgical intervention for LABC patients has been shown to yield relatively favorable survival rates. This prompts the question: can surgical treatment, in conjunction with postoperative comprehensive management, potentially cure LABC cases that were previously deemed inoperable? To address this inquiry, we have devised a prospective, single-arm cohort study aimed at assessing the feasibility, safety, and survival outcomes associated with surgical treatment for locally advanced breast cancer.

## 2 Methods

We conducted a prospective, single-arm cohort study aimed at evaluating the feasibility of administering local therapy, subsequently followed by postoperative adjuvant therapy, for the management of locally advanced breast cancer with cutaneous involvement. The ethical approval for this study was granted by the Ethics Committee of 10.13039/501100008235Peking Union Medical College Hospital (10.13039/501100008235PUMCH), under the ethical approval number I-23PJ1462. Prior to enrollment, all patients provided informed consent, ensuring adherence to ethical principles and standards within the medical research field.

Inclusion criteria: 1) 18-75 year-old woman; 2) Pathological or clinical diagnosis of breast cancer; 3) Tolerable for surgery with Eastern Cooperative Oncology Group (ECOG) Performance Status Grade 0 to 2; 4) Radiological assessment or PET evaluation showed no distant metastasis before surgery; 5) Stage T4 tumor with or without neoadjuvant treatment; 6) Volunteer to accept local modified radical surgery and complete postoperative comprehensive treatment for breast cancer; 7) Willing to receive postoperative quality of life and survival related follow-up; Exclusion criteria: 1) Pregnancy; 2) History of breast cancer or other malignant tumors; 3) Inflammatory breast cancer; 4) Not eligible for general anesthesia and surgery; 5) Unable to complete comprehensive postoperative management and follow-up.

**Fig. 1:**
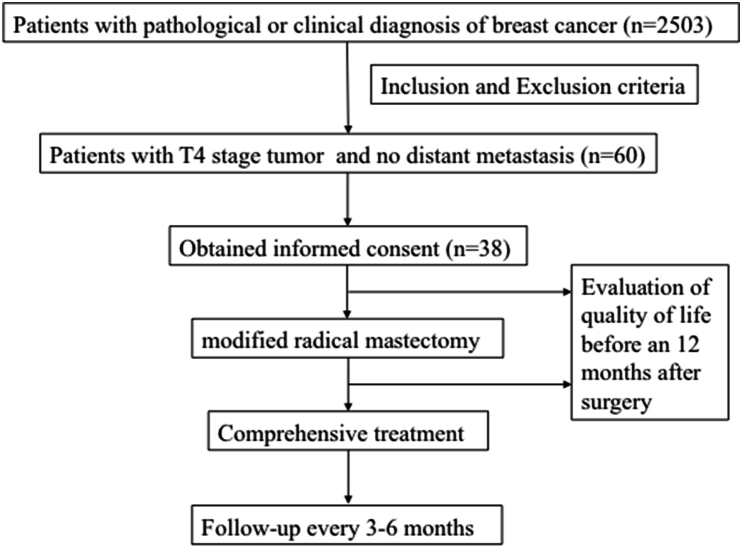
Flow chart of enrolled patients.

After signing informed consent, patients were all registered for the study preoperatively. The primary end point was overall survival (OS) and disease-free survival (DFS), and the secondary end point was the quality of life of breast cancer patients. The flow chart of enrolled patients is shown in Fig. 1. All patients enrolled received modified radical mastectomy for breast cancer in PUMCH. All patients underwent standard regimen chemotherapy including paclitaxel and anthracycline. Radiotherapy were performed after chemotherapy. Triple negative breast cancer patients received modified radical surgery, and then received standard chemotherapy, radiotherapy and subsequent capecitabine intensive treatment. Patients with HER-2 gene amplification received anti-HER-2 therapy with trastuzumab and pertuzumab, while patients with hormone receptor positive received endocrine therapy. Follow-up were performed every 3–6 months during the first 2 years after surgery, and every 6–12 months thereafter. European Organisation for Research and Treatment of Cancer (EORTC) QoL Questionnaire (QLQ) C30 core questionnaire and the EORTC QLQ-BC23 questionnaire for breast cancer patients was used to evaluate quality of life before surgery and 12 months after surgery. The EORTC QLQ-C30 (v 3.0) and EORTC QLQ B23 scales and single items were linearly transformed to 0 to 100 and analyzed according to the recommendations of the EORTC QoL Group. Self-reported Chronic lymphedema was recorded.

Categorical variables have been summarized in terms of counts, while continuous data have been presented using their average values. Kaplan-Meier curves, along with their corresponding estimates, have been provided for both disease-free survival (DFS) and overall survival (OS). Multivariate analyses were conducted utilizing a Cox proportional hazards regression model. The evaluation of quality of life was subject to comparative analysis using the paired-samples *t*-test. All statistical tests employed were two-sided, with statistical significance set at a level of 0.05. The analyses were performed utilizing R software（version 4.2.2）

## 3 Results

A total of 38 eligible patients were recruited into the study spanning from March 2018 to December 2022, with survival data followed up until March 2023. Among these patients, 19 completed quality of life assessments both preoperatively and 12 months post-surgery. The median follow-up duration was 30 months. The demographic profiles and tumor characteristics of the participants are concisely presented in Table 1. In summary, the mean age of the enrolled patients was 55.11 years. With regards to menstrual status, 44.74 % of the patients were premenopausal, while 55.26 % were postmenopausal. In terms of nodal involvement, 47.36 % of the patients presented with N3 disease, and only 13.16 % were lymph node-negative. In terms of tumor biology, 57.89 % of the tumors were hormone receptor-positive, 23.68 % were HER2-positive, nearly half belonged to the Luminal B subtype, and 28.95 % exhibited a basal-like phenotype.

**Table 1:** Patient demographics and tumor characteristics.

	All eligible patients(n = 38) (%)
Age	
Mean ± SD	55.11 ± 12.33
Menopausal Status	
Premenopausal	17(44.74)
Postmenopausal	21(55.26)
Neoadjuvant chemotherapy	
Yes	13(34.21)
No	25(65.79)
T stage	
IV	38(100)
N stage	
0	5(13.16)
I	4(10.53)
II	11(28.95)
III	18(47.36)
TNM stage	
III	38(100)
ER	
Negative	16(42.11)
Positive	22(57.89)
PR	
Negative	22(57.89)
Positive	16(42.11)
HER-2	
Negative	29(76.32)
Positive	9(23.68)
Ki-67	
<14 %	7(18.42)
≥14 %	31(81.58)
Intrinsic Molecular Subtype	
Luminal A	5(13.15)
Luminal B	17(44.74)
HER2 enriched	5(13.16)
Basal-like	11(28.95)
Chemotherapy	
Yes	38(100)
Radiotherapy	
Yes	30(78.95)
No	8(21.05)
Anti-HER2 therapy	
Yes	9(23.68)
No	29(76.32)
Endocrine therapy	
Yes	22(57.89)
No	16(42.11)

**Fig. 2:**
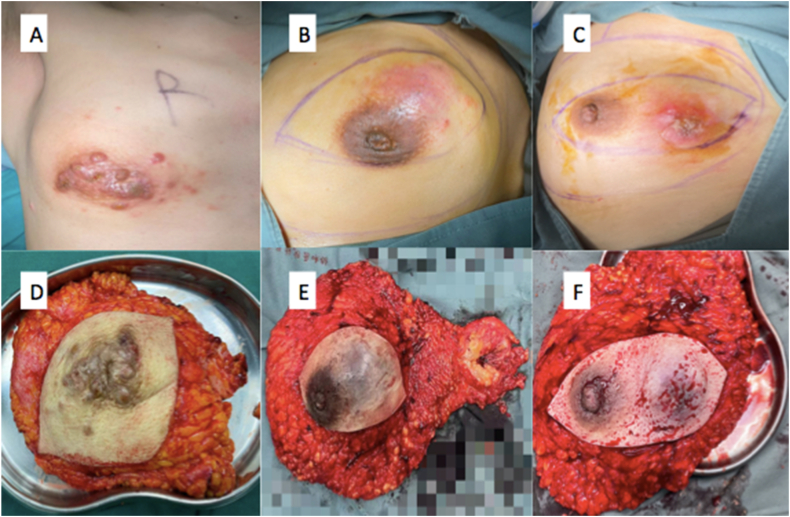
The preoperative local tumor (A–C) and intraoperative resection specimen (D–F) of three selected cases.

Prior to enrollment, 34.21 % of patients had undergone ineffective neoadjuvant chemotherapy, whereas 65.79 % of patients remained untreated following their initial diagnosis. Out of the 13 patients administered neoadjuvant chemotherapy, the efficacy assessment revealed that 61.5 % experienced progressive disease (PD), and 38.5 % had stable disease (SD). Subsequently, all patients underwent modified radical mastectomy for breast cancer and received standardized postoperative adjuvant therapy. Fig. 2(2A-2F) presents preoperative local tumor images alongside intraoperative resection specimens from three select cases, illustrating the surgical intervention and specimen characteristics.

**Fig. 3:**
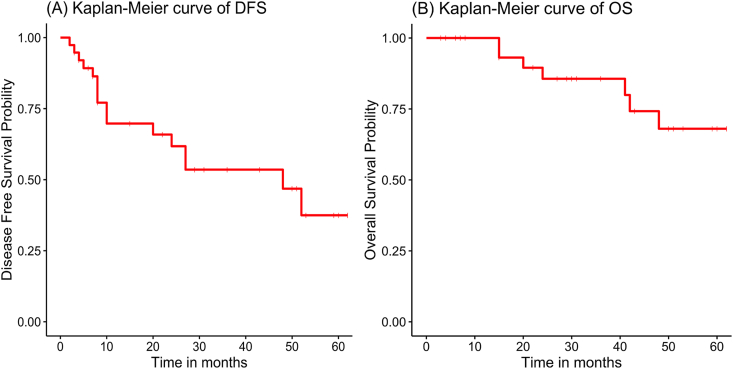
Kaplan-Meier estimated prognosis of enrolled patients: (A) Kaplan-Meier curve of Disease Free Survival(DFS) of LABC; (B) Kaplan-Meier curve of Overall Survival(OS) of LABC.

**Fig. 4:**
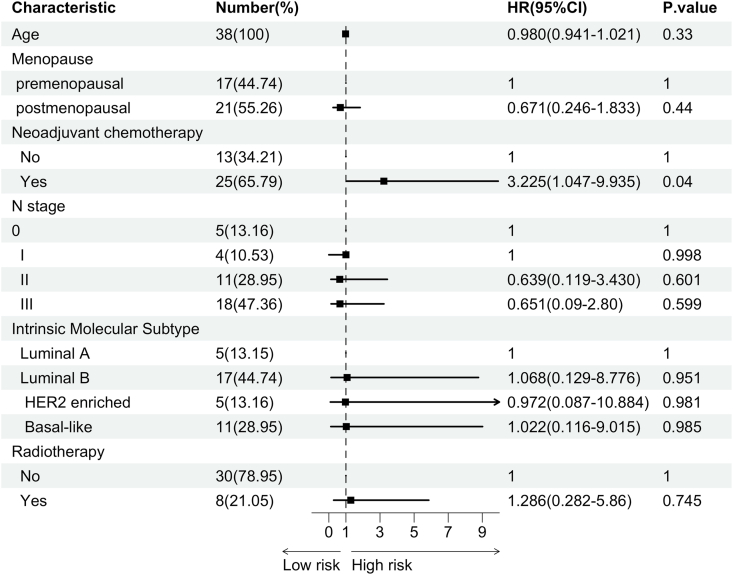
Forest plot of Univariable analysis of Disease Free Survival(DFS).

**Fig. 5:**
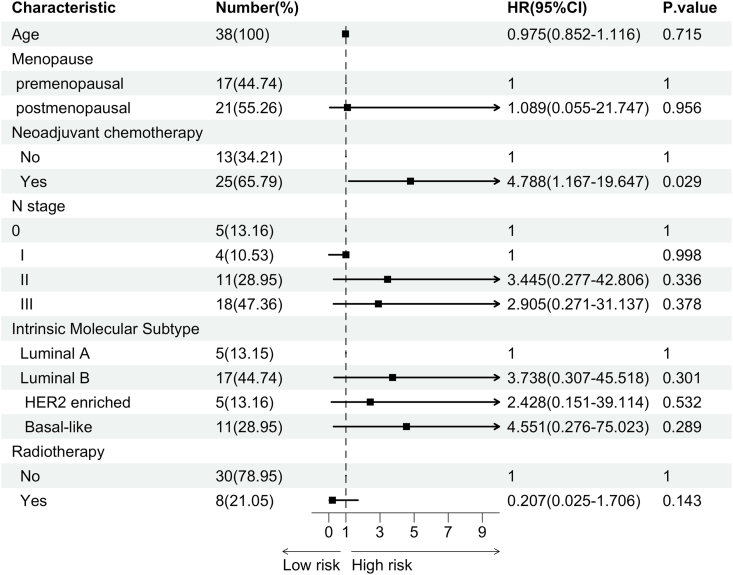
Forest plot of Multivariable analysis of Disease Free Survival(DFS).

Local recurrence was observed in 4 patients, while bone metastases occurred in 4, lung metastases in 2, liver metastasis in 1, and multiple metastases were detected in 5 patients. Additionally, 2 patients developed a second primary tumor subsequent to their breast cancer diagnosis. During the follow-up period, 7 patients succumbed to their illness. The Kaplan-Meier estimates for 1-year, 3-year, and 5-year disease-free survival (DFS) among all patients were 69.8 %, 53.3 %, and 37.5 %, respectively, while the corresponding overall survival (OS) rates were 100.0 %, 85.6 %, and 68.0 % (Table 2). The Kaplan-Meier curves illustrating DFS and OS are depicted in Fig. 3. Given the insufficient number of OS events, univariable and multivariable analyses (Fig. 4, Fig. 5) were confined to DFS. Both univariable and multivariable analyses indicated that patients with a history of neoadjuvant chemotherapy who experienced stable or progressive disease had inferior DFS outcomes. Conversely, age, menopausal status, axillary lymph node stage, and radiotherapy were not significantly associated with poor DFS.

**Table 2:** Kaplan-Meier estimated 1–5 years outcomes of DFS and OS.

	DFS		OS	
	Patients at Risk(n)	Survival Rate(%)	Patients at Risk(n)	Survival Rate(%)
1-year Survival	21	69.8(55.5–87.7)	29	100
3-year Survival	15	53.5(37.8–75.7)	23	85.6(73.5–99.8)
5-year Survival	5	37.5(20.2–69.5)	12	68.0(50.2–92.1)

During the 12-month follow-up period, 19 patients who experienced disease-free survival (DFS) or overall survival (OS) events completed quality of life assessments both preoperatively and 12 months post-surgery. Based on the EORTC QLQ-C30 scores, these patients exhibited clinically meaningful and statistically significant improvements in overall quality of life, physical functioning, role functioning, emotional functioning, and select symptom domains (P < 0.05). However, no notable improvements were observed in financial difficulties or symptom areas encompassing dyspnea, nausea/vomiting, and diarrhea (Table 3). Furthermore, the EORTC QLQ-BR23 evaluation revealed improvements in the majority of symptom and functional domains, with the exception of body image and breast-specific symptoms (Table 4). These findings underscore the positive impact of the surgical intervention and subsequent care on various aspects of patients' quality of life, while also highlighting areas where further support or intervention may be warranted.

**Table 3:** QOL Score of patients before and 12 months after surgery of EORTC QLQ-30.

Domain	Before surgery		12 months after surgery		Paried samples T test	*P* value
	Mean	SD	Mean	SD		
Quality of Life (QL)	53.95	12.79	66.67	12.97	−4.78	<0.05
Physical Functioning (PF)	67.37	17.82	89.82	11.52	−7.76	<0.05
Role Functioning (RF)	49.12	16.64	75.44	8.32	−6.76	<0.05
Emotional Functioning (EF)	57.46	22.44	82.60	16.33	−4.97	<0.05
Cognitive Functioning (CF)	58.77	24.40	88.60	10.89	−6.60	<0.05
Social Funcioning (SF)	72.81	21.77	80.71	17.32	−0.97	0.343
Fatigue (FA)	38.01	26.57	3.51	10.23	5.93	<0.05
Nausea/Vomiting (NV)	4.39	7.34	6.14	8.04	−0.697	0.494
Pain (PA)	26.32	9.85	7.02	8.23	13.47	<0.05
Dyspnoea (DY)	1.75	7.44	1.75	7.44	0	1
Insomnia (SL)	43.86	24.31	17.54	16.64	4.82	<0.05
Appetite Loss (AP)	24.56	18.23	14.04	16.46	2.88	<0.05
Constipation (CO)	8.77	21.20	7.02	17.37	0.57	0.578
Diarrhea (DI)	1.75	7.44	5.26	12.15	−1.45	0.163
Financial Problems (FI)	35.09	13.12	40.35	29.77	−0.678	0.51

**Table 4:** QOL Score of patients before and 12 months after surgery of EORTC QLQ-BR23.

Domain	Before surgery		12 months after surgery		T test	*P* value
	Mean	SD	Mean	SD		
Body image (BRBI)	56.14	20.38	62.28	24.83	−1.27	0.221
Sexual functioning (BRSEF)	73.68	15.59	94.74	12.15	−4.61	<0.05
Sexual enjoyment (BRSEE)	66.67	24.18	94.73	16.27	−4.80	<0.05
Future perspective (BRFU)	64.91	17.01	80.70	24.93	−2.45	<0.05
Systemic therapy side effects (BRST)	26.82	9.55	9.77	10.42	6.41	<0.05
Breast symptoms (BRBS)	18.42	23.35	14.47	15.49	1.06	0.305
Arm symptoms (BRAS)	28.07	12.67	19.88	10.56	3.07	<0.05
Upset by hair loss (BRHL)	31.58	7.44	14.03	16.46	3.75	<0.05

## 4 Discussion

Locally advanced breast cancer (LABC) represents an anatomical classification that encapsulates a diverse array of tumors characterized by varying presentations, biological profiles, and clinical behaviors [5,7]. In 1998, Hortobagyi et al. seminally demonstrated that a multidisciplinary approach, encompassing preoperative induction chemotherapy, surgical intervention, and radiation therapy for non-inflammatory stage III breast cancer, conferred favorable prognoses and enhanced both local and systemic disease control [8]. Prior investigations in stage III breast cancer patients have consistently shown that neoadjuvant chemotherapy, followed by surgery, radiotherapy, or a combination thereof, leads to improved DFS and OS [7]. According to the National Comprehensive Cancer Network (NCCN) guidelines, stage IIIB and IIIC breast cancers are classified as inoperable, with patients presenting with T4 tumors often identified as candidates for preoperative systemic therapy [9]. While previous research has indicated that disease progression during NCT is uncommon, there exists a subset of patients who fail to respond to neoadjuvant therapy, resulting in local tumor progression, including skin involvement [10,11]. Given the significant tumor burden, potential skin involvement, and extensive axillary lymph node metastases in these patients, disease progression can lead to loss of surgical eligibility due to the onset of metastases, ultimately compromising the opportunity for radical treatment. In our study, 13 patients underwent NCT prior to enrollment. Notably, after NCT, these patients did not experience a reduction in tumor size and, in fact, exhibited local progression, with some cases involving skin invasion.

Local surgical management of tumors holds significant clinical importance. Without it, tumors can undergo local ulceration, develop infections, and invade the chest wall, thereby predisposing patients to experiencing pain, bleeding, and upper limb lymphedema, all of which profoundly compromise their quality of life. Tumors categorized as IIIB and IIIC are relatively large and frequently involve axillary lymph nodes. Notably, in the absence of distant metastasis, locally advanced breast cancer (LABC) remains amenable to curative interventions. The surgical treatment of LABC has been the subject of limited research. A Brazilian study investigating operable LABC cases subjected to mastectomy reported a specific disease-free survival rate of 74.7 % at 60 months [12]. In contrast, a US-based study indicated a 15-year overall survival rate of 23 % for LABC patients undergoing comprehensive treatment for stage IIIB disease [13]. The Surveillance, Epidemiology, and End Results (SEER) database for IIIB/IIIC breast cancer patients reveals a 5-year breast cancer-specific survival rate ranging from 60 % to 65 % [14]. Furthermore, another study stratified 5-year survival rates according to primary tumor size, reporting 16 % and 36 % for tumors exceeding 10 cm and 5–10 cm in diameter, respectively [15]. In our study, the Kaplan-Meier estimates for 1-year, 3-year, and 5-year overall survival (OS) were 100.0 %, 85.6 %, and 68.0 %, respectively. These survival outcomes are congruent with those reported in prior investigations. Our findings indicate that the initial choice of surgical treatment in locally advanced breast cancer (LABC) does not adversely impact patient prognosis. Specifically, we observed local recurrence in 4 patients, bone metastasis in 4, lung metastasis in 2, liver metastasis in 1, and multiple metastases in 5 patients. These data align with published literature demonstrating that LABC patients frequently develop metastatic diseases [3]. In our study, the Kaplan-Meier estimates for 1-year, 3-year, and 5-year overall survival (OS) were 100.0 %, 85.6 %, and 68.0 %, respectively. These survival outcomes are congruent with those reported in prior investigations. Our findings indicate that the initial choice of surgical treatment in locally advanced breast cancer (LABC) does not adversely impact patient prognosis. Specifically, we observed local recurrence in 4 patients, bone metastasis in 4, lung metastasis in 2, liver metastasis in 1, and multiple metastases in 5 patients. These data align with published literature demonstrating that LABC patients frequently develop metastatic diseases [16]. Previous literature has highlighted that the prognostic factors in LABC are comparable to those in early-stage breast cancer, with lymph node involvement being the paramount determinant [17]. However, our study found no significant associations between poor DFS and age, menopausal status, axillary lymph node stage, or radiotherapy, likely due to the relatively smaller sample size.

In the context of quality of life (QOL) assessment, our study observed clinically meaningful and statistically significant enhancements in functional status and overall QOL among the patients. However, no noteworthy improvement was discerned in the symptomatic domain, including breast-related symptoms, dyspnea, nausea/vomiting, and diarrhea. This observation may be attributed to the fact that these symptoms in breast cancer patients with T4 tumors may not be overt, prompting them to seek medical attention at a later stage. Regarding the evaluation of QOL improvement following surgical intervention for stage IV breast cancer, a prior study has reported that surgical treatment did not confer a QOL benefit to patients [18]. Conversely, another study suggested that patients undergoing surgery for stage IV breast cancer experienced clinically significant and statistically meaningful improvements on the Global Health Status scale and the breast symptoms scale (P < 0.05) [19]. Thus, the question of whether surgical treatment augments QOL in advanced breast cancer patients remains an ambiguous issue.

This study prospectively enrolled 38 patients diagnosed with locally advanced breast cancer featuring T4 tumors. The primary objective was to evaluate the feasibility of locally modified radical resection for these cases, particularly those involving skin invasion. While the study offers valuable insights, it is important to acknowledge its inherent limitations. Firstly, the study design was unicentric and non-comparative, lacking a control group of patients who underwent surgery subsequent to standard neoadjuvant chemotherapy. This restricts the generalizability of the findings. Secondly, being an observational study, it does not enforce uniform standards for adjuvant therapy administration among participants. Notably, 8 patients did not receive postoperative radiotherapy, whereas the remaining cohort completed the standard treatment protocol. Thirdly, the patient population was selected based on their overall good health status and willingness to undergo both surgery and subsequent adjuvant treatments, potentially introducing a selection bias into the study. Lastly, the follow-up duration for patients remains relatively short, with only 5 patients having completed over 60 months of follow-up. To accurately assess the long-term prognosis of locally advanced breast cancer patients undergoing surgical intervention, a further extension of the follow-up period is warranted.

## 5 Conclusion

Our study demonstrated that patients with locally advanced breast cancer harboring T4 tumors who undergo surgical resection followed by postoperative adjuvant therapy can attain favorable prognosis and an enhanced quality of life. Surgical intervention, as an initial treatment modality, merits consideration for patients with locally advanced breast cancer. This approach offers a viable option that aligns with the aim of optimizing patient outcomes and preserving quality of life. Our study is a single-center observational study that involves a relatively modest sample size of enrolled patients and a limited duration of follow-up. Consequently, the research findings inherently possess certain limitations. To enhance the robustness and validity of our conclusions, future endeavors should incorporate randomized controlled trials (RCTs) with a longer follow-up period, thereby allowing for a more comprehensive validation of the research outcomes. This approach will facilitate a deeper understanding of the phenomenon under investigation and contribute to the advancement of medical knowledge in this field.

## Funding/support

Supported by National High Level Hospital Clinical Research Funding (2022-PUMCH-C-066) as a non-restricted grant.

## Ethics statement

The ethical approval for this study was granted by the Ethics Committee of 10.13039/501100008235Peking Union Medical College Hospital (10.13039/501100008235PUMCH), under the ethical approval number I-23PJ1462. Prior to enrollment, all patients provided informed written consent.

## Data availability statement

Data will be made available on request.

## CRediT authorship contribution statement

**Ying Xu:** Writing – original draft, Project administration, Methodology, Data curation. **Xin Huang:** Writing – review & editing, Investigation, Formal analysis, Data curation. **Chang Chen:** Writing – review & editing, Methodology, Data curation. **Yan Li:** Supervision, Formal analysis, Data curation. **Yidong Zhou:** Supervision, Methodology, Investigation. **Songjie Shen:** Supervision, Methodology, Investigation, Data curation. **Yan Lin:** Writing – review & editing, Supervision, Methodology, Investigation, Funding acquisition, Data curation. **Qiang Sun:** Writing – review & editing, Validation, Methodology, Data curation.

## Declaration of competing interest

The authors declare that they have no known competing financial interests or personal relationships that could have appeared to influence the work reported in this paper.
